# Revolutionizing Reproduction: The Impact of Robotics and Artificial Intelligence (AI) in Assisted Reproductive Technology: A Comprehensive Review

**DOI:** 10.7759/cureus.63072

**Published:** 2024-06-24

**Authors:** Smruti A Mapari, Deepti Shrivastava, Gautam N Bedi, Utkarsh Pradeep, Aman Gupta, Paschyanti R Kasat, Pratiksha Sachani

**Affiliations:** 1 Obstetrics and Gynaecology, Jawaharlal Nehru Medical College, Datta Meghe Institute of Higher Education and Research, Wardha, IND; 2 Medicine, Jawaharlal Nehru Medical College, Datta Meghe Institute of Higher Education and Research, Wardha, IND; 3 Radiodiagnosis, Jawaharlal Nehru Medical College, Datta Meghe Institute of Higher Education and Research, Wardha, IND

**Keywords:** precision medicine, reproductive medicine, infertility, artificial intelligence (ai), robotics, assisted reproductive technology (art)

## Abstract

Assisted reproductive technology (ART) has revolutionized the field of reproductive medicine, offering hope to millions of individuals and couples facing infertility challenges. In recent years, integrating robotics and artificial intelligence (AI) has emerged as a promising avenue for advancing ART. This comprehensive review explores the transformative impact of robotics and AI on ART, examining recent advancements, technological applications, clinical implications, and ethical considerations. Robotics enables precise and minimally invasive procedures, enhancing the efficiency and accuracy of various reproductive techniques such as sperm retrieval, embryo handling, and surgical interventions. Meanwhile, AI offers predictive analytics, personalized treatment protocols, and decision support systems tailored to individual patient needs, optimizing treatment outcomes and expanding access to reproductive care. Key findings highlight the significant advancements made possible by robotics and AI in ART, including improved success rates, reduced risks, and enhanced patient experience. However, challenges such as regulatory considerations, adoption barriers, and ethical dilemmas must be addressed to realize the full potential of these technologies. The transformative impact of robotics and AI on ART is profound, shaping the future of fertility treatment and family-building worldwide. Continued research, interdisciplinary collaboration, and investment are essential to further harness the potential of robotics and AI in advancing reproductive medicine and ensuring accessible, equitable, and effective care for all individuals and couples.

## Introduction and background

Assisted reproductive technology (ART) comprises a range of medical procedures used to facilitate conception when natural conception is difficult or impossible [1]. These techniques typically involve the manipulation of sperm, eggs, or embryos outside of the body to achieve a successful pregnancy. ART has revolutionized the field of reproductive medicine, offering hope to millions of individuals and couples facing infertility issues worldwide [2].

In recent years, integrating robotics and artificial intelligence (AI) has emerged as a promising avenue for advancing ART [3]. Robotics allows for precise and minimally invasive procedures, enhancing the efficiency and accuracy of various reproductive techniques such as sperm retrieval, embryo handling, and surgical interventions. Meanwhile, AI enables data analysis, predictive modeling, and decision support, offering insights that can optimize treatment protocols, improve success rates, and personalize care for patients undergoing ART procedures [4].

This comprehensive review aims to explore the transformative impact of robotics and AI on ART. By examining recent advances, technological applications, clinical implications, and ethical considerations, this review aims to thoroughly understand how robotics and AI are reshaping the landscape of reproductive medicine. Additionally, the review will highlight the emerging trends, challenges, and future directions in the field, aiming to inform clinicians, researchers, policymakers, and other stakeholders about the potential benefits and complexities associated with integrating robotics and AI into ART practice.

## Review

Historical perspective of assisted reproductive technology

Milestones in the Development of ART

In 1978, the landscape of reproductive medicine changed forever with the birth of Louise Brown, heralded as the first "test-tube baby." This historic event marked the advent of in vitro fertilization (IVF) as a groundbreaking solution for infertility, offering hope to countless individuals and couples struggling to conceive [5]. The 1980s witnessed the diversification of assisted reproductive technology (ART) techniques with the introduction of alternative approaches such as gamete intrafallopian transfer (GIFT) and zygote intrafallopian transfer (ZIFT). These methods expanded the options available to patients seeking fertility treatments, contributing to the evolving landscape of reproductive medicine [6]. Advancements in the 1990s propelled ART forward, addressing specific challenges in infertility treatment. Intracytoplasmic sperm injection (ICSI) emerged as a revolutionary technique for overcoming male infertility. At the same time, preimplantation genetic diagnosis (PGD) offers a means of screening embryos for genetic disorders, enhancing the selection process and minimizing risks associated with genetic abnormalities [7]. The early 2000s witnessed significant progress in embryology laboratory techniques and cryopreservation methods. Improvements in vitrification techniques for cryopreserving embryos and oocytes revolutionized the field, significantly improving success rates for ART procedures and offering new possibilities for patients undergoing fertility treatments [6]. From 2005 to 2013, ART experienced further advancements, including adopting controlled ovarian hyperstimulation and luteal phase support. Additionally, innovative technologies such as embryonic genetic testing and oocyte preservation emerged, expanding the scope of possibilities for patients undergoing fertility treatments and enhancing the effectiveness of ART procedures [6]. In the current era, research and innovation in ART continue unabated. Ongoing studies explore novel approaches such as minimal stimulation protocols and gonadotropin-releasing hormone (GnRH) agonist cycle triggers, aiming to optimize treatment outcomes and improve the patient experience. Furthermore, applying metabolomics and proteomics holds promise for refining IVF outcomes, underscoring the dynamic nature of reproductive medicine and the relentless pursuit of excellence in fertility care [6].

Challenges Faced by Traditional ART Methods

**Figure 1:**
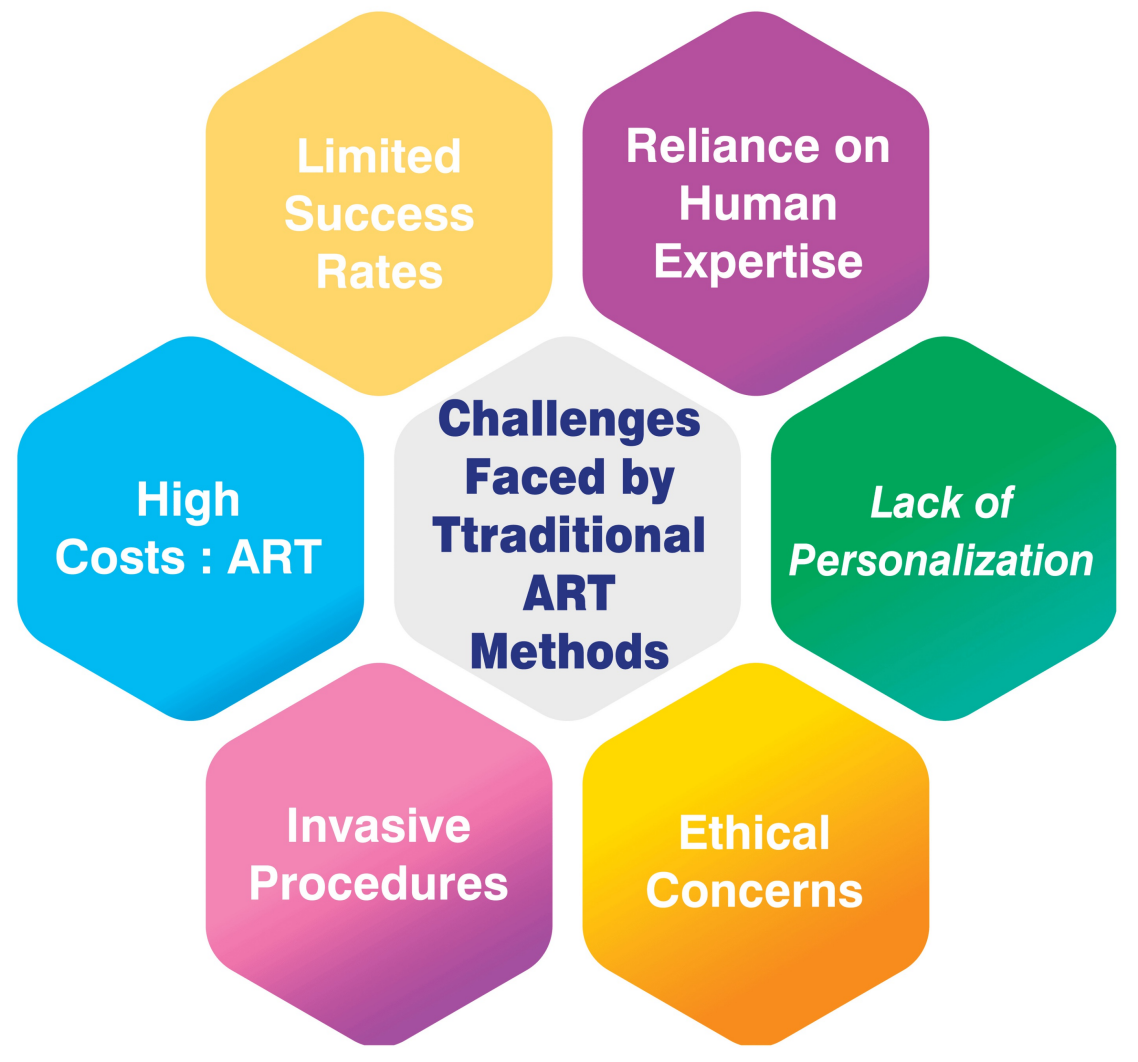
Challenges faced by traditional ART methods Image credit: Dr. Smruti A. Mapari

Traditional ART methods, such as in vitro fertilization and intracytoplasmic sperm injection, face challenges that limit their efficacy. One significant hurdle is their relatively low success rates, typically ranging from 20% to 30% live birth rates per embryo transfer [8,9]. Additionally, the high costs associated with ART procedures pose a barrier to access, with the average price of a single IVF cycle in the United States being $12,000-$15,000, rendering treatment financially out of reach for many infertile couples [8,9]. Moreover, traditional ART techniques often entail invasive procedures like egg retrieval and embryo transfer, which can take a physical and emotional toll on patients [8,9]. These invasive procedures, coupled with the uncertainty of success, contribute to the burdens faced by individuals undergoing fertility treatments. Ethical considerations also loom large in the realm of ART, touching upon issues such as embryo selection, the risk of multiple births, and the ethical dilemmas surrounding the fate of unused embryos [8,9]. The complex ethical landscape complicates the decision-making process for patients and healthcare providers. Furthermore, traditional ART protocols typically adopt a one-size-fits-all approach, neglecting to account for individual patients' unique characteristics and preferences [8,9]. This lack of personalization may limit the effectiveness of treatment and overlook critical factors that could influence outcomes. Another challenge is the heavy reliance on human expertise in current ART methods, which introduces variability in outcomes and increases the potential for human error [8,9]. The subjective nature of human judgment in embryology and clinical practice underscores the need for more standardized and technologically driven approaches to enhance the reliability and consistency of ART procedures. The challenges faced by traditional ART methods are shown in Figure 1.

Need for Innovation in Reproductive Technology

In the realm of reproductive technology, there exists a pressing demand for innovation aimed at enhancing effectiveness, alleviating burdens, and ensuring equitable access to assisted reproduction techniques. The introduction of novel reproductive technologies often occurs without a thorough assessment of their efficacy, safety, and impact on patients and children born. Responsible innovation entails subjecting potentially risky reproductive technologies to rigorous research, including preclinical investigations, clinical trials, and long-term follow-up studies, to guarantee their safety and efficacy [10]. Innovations such as preimplantation genetic testing (PGT), time-lapse imaging, mitochondrial replacement therapy (MRT), artificial intelligence (AI) algorithms for predicting IVF success, and in vitro maturation (IVM) of eggs are revolutionizing the ART landscape. These advancements are designed to enhance success rates, mitigate genetic abnormalities, refine embryo selection processes, and provide alternative options for individuals grappling with infertility challenges [11]. The history of assisted reproductive technology has witnessed remarkable progress, from the initial experiments in artificial insemination to the milestone birth of the first "test-tube baby" through IVF. With ongoing advancements in technology and medical knowledge, the field of reproductive medicine stands on the cusp of further breakthroughs that promise to render infertility treatments safer, more efficient, and more accessible to a broader spectrum of patients [11,12].

Robotics in assisted reproductive technology

Robotic Systems Used in Reproductive Medicine

Robotic-assisted laparoscopic surgery has emerged as a viable alternative to traditional laparoscopic techniques for a range of procedures, including tubal reanastomosis, myomectomy, endometriosis surgery, and ovarian transplantation [13-15]. These robotic systems offer enhanced precision, dexterity, and visualization capabilities, resulting in superior outcomes and expedited recovery times for patients [13-15]. In ART labs, robotic systems are pivotal in automating and optimizing processes such as embryo culture, tracking, and freezing [13-15]. By minimizing errors, improving record-keeping, and enhancing overall efficiency, these systems are revolutionizing in vitro fertilization procedures [13,15]. Exploration is underway into robotic systems for the automated selection and injection of sperm cells during intracytoplasmic sperm injection procedures. This potential application holds promise for standardizing and streamlining sperm selection, potentially enhancing fertilization rates [13]. Additionally, robotic systems are being investigated for the automated removal of cumulus cells from oocytes, known as oocyte denudation, before intracytoplasmic sperm injection. By reducing the risk of human error and ensuring consistency in this critical step, these systems aim to improve the overall efficiency of ART procedures [13]. Integrating robotic systems into reproductive medicine represents an evolving frontier with substantial potential for further advancements [13-15]. As these technologies progress, they are poised to revolutionize various facets of reproductive medicine, from surgical interventions to laboratory processes. Ultimately, these innovations promise to deliver improved outcomes and enhanced patient care for individuals grappling with infertility [13].

Advantages of Robotics Over Conventional Techniques

The integration of robotics into reproductive surgery presents numerous advantages over conventional techniques. Procedures such as robotic-assisted hysterectomy in reproductive surgery offer a host of benefits, including diminished pain, decreased blood loss, accelerated recovery periods, and shorter hospital stays in comparison to traditional open surgeries [16]. Robotic technology amplifies surgical precision and facilitates a broader range of motion, resulting in superior outcomes and potentially fewer complications [16]. Furthermore, robotic surgery provides high-definition, three-dimensional visualization of the surgical site, empowering surgeons to execute procedures with heightened accuracy and control [16]. Additionally, robotic-assisted surgeries are minimally invasive, involving smaller incisions that can yield reduced scarring, diminished infection risk, and expedited post-operative healing for patients [16]. Using robotics in reproductive surgeries such as tubal reanastomosis, myomectomy, endometriosis, and ovarian transplantation underscores the potential for remote surgery, precision, and enhanced patient outcomes [4,15,17]. In summary, the benefits of robotics in reproductive surgery encompass augmented precision, diminished invasiveness, hastened recovery periods, and improved patient outcomes when juxtaposed with traditional surgical methodologies.

Examples of Robotic Applications in ART Procedures

Robotic-assisted sperm retrieval represents a significant advancement within the realm of ART, showcasing the successful integration of robotic technology into various procedures. Particularly in the field of andrology, robotic-assisted microsurgery has demonstrated promising outcomes, notably in procedures like vasectomy reversal. Research has underscored the advantages of robotic assistance in vasectomy reversal surgeries, illustrating shorter operative times, comparable setup times to traditional microsurgery, and eliminating the necessity for a skilled microsurgical assistant due to the additional robotic arm [18]. Furthermore, robotic systems have proven valuable in circumventing fibrotic changes in cases of iatrogenic vasal injuries, as well as in procedures such as varicocelectomy and microsurgical denervation of the spermatic cord, resulting in enhancements in sperm parameters and pain management [18]. The utilization of robotics in sperm retrieval procedures, encompassing vasovasostomy and vasoepididymostomy, has yielded favorable outcomes, with a significant percentage of cases experiencing pain alleviation and reduced operative times [18]. These advancements in robotic technology are elevating the precision, efficiency, and outcomes of ART procedures, ushering in new avenues for male infertility treatments.

Robotic applications in ART procedures, specifically in robotic embryo handling and manipulation, demonstrate considerable promise in augmenting the efficiency and precision of various processes. One noteworthy application is the deployment of robotic systems for embryo culture, tracking, and freezing, which has resulted in enhanced outcomes and diminished errors in procedures such as in vitro fertilization [19-21]. Additionally, robotic technology has been incorporated into surgeries pertinent to reproductive medicine, such as tubal reanastomosis, myomectomy, endometriosis surgery, and ovarian transplantation, showcasing the potential for remote surgery and meticulous procedures in fertility preservation [21]. Moreover, integrating robotics in ART laboratories has streamlined tasks like embryo selection, transfer, and witnessing, offering advantages such as heightened reproducibility, minimal error rates, and enhanced record maintenance in ART procedures [19-21]. These advancements underscore the transformative influence of robotics in ART, paving the way for more efficient and effective infertility treatments with the potential to revolutionize the field of reproductive medicine.

Robotic-assisted surgery is emerging as a valuable technology in reproductive medicine, offering an alternative to traditional surgical techniques. It finds application in various surgeries including tubal reanastomosis, myomectomy, endometriosis surgery, and ovarian transplantation, demonstrating the potential for remote surgery and precision of the procedure [13,20]. Furthermore, robotics are employed in procedures aimed at fertility preservation, such as tubal reanastomosis, ovarian transposition, radical trachelectomy, and ovarian transplantation, utilizing minimally invasive approaches to enhance the restoration and preservation of fertility in select patients [20]. In ART lab processes, robotics streamline tasks like embryo culture, tracking, and freezing, leading to improved outcomes, reduced errors, and enhanced record maintenance in procedures like in vitro fertilization [13,16,20]. These diverse robotics applications in ART procedures underscore the potential for heightened precision, efficiency, and outcomes in reproductive medicine, paving the way for a more advanced and practical approach to assisted reproduction.

Artificial intelligence in assisted reproductive technology

Role of AI in Improving Success Rates of ART Procedures

Artificial Intelligence (AI) plays a pivotal role in elevating the success rates of ART procedures, particularly in vitro fertilization (IVF) [4,22-24]. A significant impact area of AI lies in embryo selection. Historically, assessing embryo quality relied on subjective human judgment. Still, AI algorithms now enable objective analysis of embryo images, facilitating standardized scoring and potentially enhancing the selection of viable embryos for transfer [4,24]. AI-based tools can predict embryo ploidy status and implantation potential, assisting clinicians in identifying the most suitable embryos for transfer [23,24]. Additionally, AI facilitates personalized ART treatments by factoring in individual patient variables such as age, medical history, and response to previous treatments, thereby augmenting the predictive accuracy of IVF outcomes [23,24]. AI algorithms analyze patient data to craft patient-specific treatment plans, optimizing the IVF process and improving success rates [23]. Furthermore, AI integration spans various facets of the IVF procedure, encompassing sperm classification, stimulation protocol selection, oocyte quality assessment, and workflow optimization [4,24]. These advancements aim to mitigate interobserver variability, adjust drug doses during oocyte stimulation, and enhance the overall efficiency and precision of the IVF process [4,24]. However, the adoption of AI in ART presents challenges related to data privacy, algorithm bias, and ethical considerations [22,23]. Thorough testing through high-quality randomized controlled trials and external validation is imperative before AI tools can be routinely integrated into clinical decision-making processes [22,23].

AI Applications in Gamete and Embryo Selection

The application of AI in gamete and embryo selection within ART represents a rapidly evolving field with substantial potential to bolster the success rates of ART procedures. AI technology harnesses the capability to analyze vast quantities of data, particularly images and videos, facilitating more objective and accurate assessment of sperm, oocytes, and embryos. By utilizing well-trained AI models, embryologists benefit from expedited calculations and heightened precision in selecting gametes and embryos for ART treatments [25,26]. A range of AI models explicitly tailored for gamete and embryo assessment have been developed, showcasing promising performance in refining the selection process. These AI tools are pivotal in optimizing embryo ranking and selection procedures by extracting pertinent information from microscopy images, ultimately striving to pinpoint the most viable embryos with the highest likelihood of successful pregnancies [26]. Integrating AI in gamete and embryo selection revolutionizes the efficiency and precision of ART procedures and prompts significant considerations surrounding evaluation metrics, comparison methodologies, and potential biases in AI models. As the field progresses, it becomes imperative to confront these challenges to ensure the ethical and effective integration of AI technologies in reproductive medicine [26].

Predictive Analytics and Decision Support Systems in ART

AI and machine learning are fundamentally reshaping predictive analytics and decision support systems within ART, profoundly impacting the in vitro fertilization treatment process [27-29]. These advanced tools empower clinicians and embryologists to make more informed decisions, optimizing patient care and outcomes. AI-based decision support systems have been specifically crafted to streamline and automate scheduling and dose adjustments during ovarian stimulation. By accurately predicting the optimal day for triggering final oocyte maturation, these systems contribute to improved treatment outcomes and reduced costs [27,30]. Furthermore, machine learning algorithms are pivotal in forecasting live birth outcomes and early pregnancy loss following IVF-embryo transfer, furnishing clinicians with invaluable insights [27,30]. Predictive calculators leverage AI algorithms, encompassing classical machine learning, deep learning, and ensemble methods, to assess relevant parameters and furnish personalized predictions. These calculators are indispensable tools for patients and healthcare providers, establishing realistic expectations regarding the likelihood of success in ART treatments [27,30]. Moreover, AI facilitates workflow optimization during ovarian stimulation and IVF procedures. Computer algorithms tailored explicitly for IVF management demonstrate remarkable accuracy in day-to-day decision-making processes, thus providing a potential avenue to augment clinical decision-making and enhance the overall patient experience [27,30].

Ethical Considerations Surrounding the Use of AI in Reproductive Medicine

The ethical considerations surrounding the integration of AI in reproductive medicine are intricate and paramount. As AI becomes increasingly integrated into ART, many normative questions emerge, particularly concerning the evidence of efficacy, informed consent, potential risks for offspring, and the impact on patient autonomy and welfare [31-33]. One pivotal ethical concern revolves around the imperative for transparency and rigorous evaluation of AI systems employed in reproductive medicine to safeguard patient safety and well-being. These ethical challenges encompass issues of fairness in resource allocation, cost reimbursement, and various stakeholders' accountability in decision-making processes within AI-assisted reproductive procedures [31,33]. Furthermore, the ethical implications of AI in reproductive medicine encompass considerations of data privacy, algorithmic biases, and the potential social dynamics that may ensue from the widespread adoption of AI technologies in fertility treatments. Ensuring that AI applications in ART adhere to ethical principles entails critically scrutinizing the quality and quantity of data utilized, addressing transparency concerns, and proactively anticipating and mitigating any adverse impacts on patients and society [31,33].

Integration of robotics and AI in assisted reproductive technology

Synergistic Effects of Combining Robotics and AI

Integrating robotics and AI into ART heralds a transformative era in reproductive medicine. These innovations are poised to revolutionize ART procedures by enhancing efficiency, reproducibility, and consistency. Through automation facilitated by computerized systems and robotic technologies, existing processes are expected to be streamlined, costs reduced, and the accessibility and affordability of ART treatments increased [19,34,35]. One notable area where AI and robotics are exerting a significant impact is in embryo selection. Historically reliant on subjective human judgment, embryo quality assessment now benefits from AI algorithms, enabling the objective analysis of embryo images. This facilitates standardized scoring and promises to improve the selection of viable embryos for transfer [35]. Furthermore, AI facilitates personalized ART treatments by considering individual patient factors such as age, medical history, and response to previous treatments, thereby enhancing the predictive capacity of IVF outcomes [19]. The trajectory of ART is shifting towards partial automation and intelligent devices capable of streamlining the entire IVF treatment pathway, from patient consultations to gamete/embryo selection, endometrial evaluation, and cryopreservation processes. The convergence of microfluidics, AI, and robotics offers the prospect of fully automated and intelligent systems, potentially reshaping the roles of IVF professionals and bolstering the overall success rates of ART procedures [19].

Automated Systems for Monitoring and Optimizing ART Procedures

The integration of automated systems, such as time-lapse microscopy (TLM) and machine learning/AI, in embryo assessment and selection marks a significant advancement in ART. These systems facilitate continuous embryo monitoring and assist in scoring and selecting embryos. AI algorithms analyze embryo images objectively, standardizing scoring and enhancing the selection of viable embryos for transfer. By mitigating variability associated with manual embryo assessment, these systems bolster the predictive capacity of IVF outcomes [19,36]. Automated patient identification systems, exemplified by electronic witnessing systems (EWS), are crucial in accurately tracking patient specimens throughout the ART process. They minimize errors and mix-ups, providing traceability and improving supervision of patient samples. This contributes to enhanced ART laboratory practices and reduces the risk of critical mismatch errors [19]. In cryopreservation and laboratory duties, automated systems streamline processes, diminish variability, and alleviate manual workload in ART laboratories. Semi-automated vitrification systems exhibit promising outcomes, optimizing laboratory logistics and potentially improving clinical outcomes. Automation in mainstream laboratory duties enhances standardization in methodologies and results, offering a more efficient and reliable approach to ART procedures [36]. The convergence of microfluidics, AI, and robotics holds immense potential to develop fully automated and intelligent devices capable of streamlining the entire IVF treatment pathway. The integration of AI with time-lapse imaging for embryo assessment is expanding, providing a versatile and resourceful tool for embryologists and IVF laboratories. These advancements are poised to revolutionize ART practices, offering personalized strategies for embryo selection and enhancing overall success rates in assisted reproduction [19].

Future Directions and Emerging Trends in Robotic-Assisted ART

The future trajectory and emerging trends in robotic-assisted ART are characterized by substantial advancements poised to redefine the landscape of reproductive medicine. These trends encompass the integration of automation, robotics, and AI to bolster the efficiency, reproducibility, and consistency of ART procedures . The convergence of microfluidics, AI, and robotics holds the potential to usher in fully automated and intelligent systems, potentially revolutionizing the roles of IVF professionals and enhancing the overall success rates of ART procedures [19,37]. Furthermore, integrating AI technology into IVF clinics represents a frontier in propelling personalized reproductive medicine. AI stands to refine and enhance current clinical practices, augmenting the predictive capacity of IVF outcomes. This advancement is poised to benefit patients and contribute to the birth of a healthier generation conceived through IVF, particularly in light of the anticipated surge in demand for IVF services in the forthcoming years [19].

## Conclusions

In conclusion, integrating robotics and AI in ART represents a significant leap forward in reproductive medicine. Robotics offer precision and minimally invasive procedures, while AI provides data-driven insights and personalized treatment protocols. Together, these technologies hold the potential to revolutionize ART by improving success rates, optimizing patient outcomes, and expanding access to reproductive care. However, realizing this potential requires further research and interdisciplinary collaboration. Continued innovation in robotics, AI algorithms, and integration strategies is necessary to address challenges and broaden the scope of ART applications. Moreover, collaboration among reproductive endocrinologists, engineers, data scientists, ethicists, and policymakers is essential for navigating these technologies' complex ethical, legal, and social implications. By fostering collaboration and investment in research and clinical practice, we can ensure that robotics and AI contribute to more accessible, equitable, and effective reproductive care globally.
